# The International Encyclopædia of Surgery

**Published:** 1883-08

**Authors:** 


**The International Encyclopædia of Surgery,** a systematic treatise on the theory and practice of surgery by authors of various nations. Edited by John Ashurst, M. D., Professor of Chemical Surgery in the University of Pennsylvania. Illustrated with chromo-lithographs and wood-cuts. In six volumes. Vol. iii. New York: Wm. Wood & Co. 1883.

The third volume of this great work sustains the high reputation acquired by its predecessors. It is devoted to the consideration of the injuries and diseases of the various tissues of the human body, containing separate articles on injuries and diseases of muscles, tendons and fascia; on injuries and surgical diseases of lymphatics; on injuries of blood vessels; on surgical diseases of the vascular system; on aneurism; on injuries and diseases of the nerves (including tetanus), and on injuries of the joints. The authors are respectively: Andrews, Barwell, Bellamy, Conner, Lidell, Nicaise and Wyeth; and most admirably has each accomplished the work attempted. The subjects are not only treated exhaustively, but also in an eminently practical manner. The work is in happy contrast with that of Ziemsen's Encyclopædia of Medicine in its more practical value, and if the succeeding volumes are equal to the three now issued, the work will be a necessity to every surgeon. The book is admirably presented by the publishers. The illustrations are excellent and numerous.

